# Changing Patterns of Antihyperglycaemic Treatment among Patients with Type 2 Diabetes in Hungary between 2015 and 2020—Nationwide Data from a Register-Based Analysis

**DOI:** 10.3390/medicina58101382

**Published:** 2022-10-01

**Authors:** György Jermendy, Zoltán Kiss, György Rokszin, Zsolt Abonyi-Tóth, Csaba Lengyel, Péter Kempler, István Wittmann

**Affiliations:** 1 Department of Internal Medicine, Bajcsy-Zsilinszky Teaching Hospital and Outpatient Clinic, Maglódi út 89-91, 1106 Budapest, Hungary; 2 Nephrology-Diabetes Center, 2nd Department of Internal Medicine, Faculty of Medicine, University of Pécs, Pacsirta út 1, 7624 Pécs, Hungary; 3 RxTarget Ltd., Bacsó Nándor utca 10, 5000 Szolnok, Hungary; 4 Department of Internal Medicine, Faculty of Medicine, University of Szeged, Kálvária sgt. 57, 6725 Szeged, Hungary; 5 Department of Internal Medicine and Oncology, Faculty of Medicine, Semmelweis University, Korányi Sándor út 2, 1082 Budapest, Hungary

**Keywords:** diabetes therapy, type 2 diabetes, register-based analysis, antihyperglycaemic drugs, metformin, sulfonylurea, insulin treatment, DPP-4 inhibitors, SGLT-2 inhibitors, GLP-1 receptor agonists

## Abstract

*Background and objectives*: In the last couple of years, pharmacological management of patients with type 2 diabetes mellitus (T2DM) have been markedly renewed. The aim of this study was to analyse the changes in prescribing patterns of antidiabetic drugs for treating patients with T2DM in Hungary between 2015 and 2020. *Material and Methods*: In this retrospective, nationwide analysis, we used the central database of the National Health Insurance Fund. We present annual numbers and their proportion of T2DM patients with different treatment regimens. *Results*: In the period of 2015–2020, the number of incident cases decreased from 60,049 to 29,865, while prevalent cases increased from 682,274 to 752,367. Patients with metformin (MET) monotherapy had the highest prevalence (31% in 2020). Prevalence of insulin (INS) monotherapy continuously but slightly decreased from 29% to 27% while that of sulfonylurea (SU) monotherapy markedly decreased from 37% to 20%. Dipeptidyl peptidase (DPP-4) inhibitors remained popular in 2020 as monotherapy (5%), in dual combination with MET (12%) and in triple combination with MET and SU (5%). The prevalence of patients with sodium-glucose co-transporter-2 (SGLT-2) inhibitors increased from 1% to 4% in monotherapy, from <1% to 6% in dual combination with MET, and from <1% to 2% in triple oral combination with MET and SU or DPP-4-inhibitors. The prevalence of patients using glucagon-like peptide-1 receptor agonists (GLP-1-RAs) also increased but remained around 1–2% both in monotherapy and combinations. For initiating antihyperglycaemic treatment, MET monotherapy was the most frequently used regime in 2020 (50%), followed by monotherapy with SUs (16%) or INS (10%). After initial MET monotherapy, the incidence rates of patients with add-on GLP-1-RAs (2%, 3%, and 4%) and those of add-on SGLT-2 inhibitors (4%, 6%, and 8%) slowly increased in the subsequent 24, 48, and 72 months, respectively. *Conclusions*: In the period of 2015–2020, we documented important changes in trends of antihyperglycaemic therapeutic patterns in patients with T2DM which followed the new scientific recommendations but remained below our expectations regarding timing and magnitude. More efforts are warranted to implement new agents with cardiovascular/renal benefits into therapeutic management in time, in a much larger proportion of T2DM population, and without delay.

## 1. Introduction

The prevalence of type 2 diabetes mellitus (T2DM) has been continuously increasing worldwide, although this trend in the European population should be considered modest as compared to some other regions of the world. In the 9th edition of the International Diabetes Federation (IDF) Diabetes Atlas, the global diabetes prevalence was estimated to be 9.3% among the adult (age: 20–79 years) population in 2019 and the number of people with diabetes (463 million) was projected to increase by 25% in 2030 and 51% in 2045 [1]. Nevertheless, these numbers proved to be underestimated in light of recent IDF Diabetes Atlas 10th edition in 2021 [2]. 

T2DM is the most prevalent form of diabetes in the adulthood. Several epidemiological studies from different countries documented increasing trends in its prevalence rate over time in the last 2–3 decades [3,4,5,6,7]. These clear changes could be attributed to both ageing with increased life expectancy in the entire population and to new pharmacological treatment options delaying progression of the disease and to the reduced mortality rate.

As T2DM diabetes is a chronic disease, management of this metabolic disorders is of great importance. Undoubtedly, the research activity of the pharmacological industry was very active and extremely successful for developing new antihyperglycaemic agents from the beginning of the new millennium. As a result, novel antidiabetic drugs such as dipeptidyl peptidase-4 inhibitors (DPP-4 inhibitors), sodium-glucose co-transporter-2 inhibitors (SGLT-2 inhibitors), and glucagon-like peptide-1 receptor agonists (GLP-1-RAs) became available in the last couple of years, although traditional agents such as metformin (MET), sulfonylureas (SUs), and insulin (INS) remained widely used. Consequently, trends of using antihyperglycaemic agents for treating patients with T2DM began to change [8,9,10,11,12].

The changing patterns of antihyperglycaemic treatment in patients with T2DM have both general features and country-specific characteristics. The former ones are related to international therapeutical guidelines and recommendations. It became clear that SGLT-2 inhibitors and/or GLP-1-RAs should be considered as recommended drugs for patients with atherosclerotic cardiac diseases, heart failure, or chronic kidney diseases [13]. As for the country-specific characteristics, they are mainly linked to availability of a particular drug, and importantly, to national reimbursement policy. 

In Hungary, we have had data about the treatment patterns from the period of 2001–2014 but this analysis could not involve SGLT-2 inhibitors, and the use of GLP-1-RAs was limited at that time [14]. Consequently, we could not evaluate important details of the management of T2DM. Therefore, we decided to perform a new analysis about the patterns of treatment with antihyperglycaemic agents in patients with T2DM to obtain updated and more detailed results. Using the central database of the National Health Insurance Fund, we were able to perform a nationwide analysis in the period of 2015–2020. 

## 2. Material and Methods

We used the database of the National Institute of Health Insurance Fund (Hungary) (study license number: I043/35-3/2021). In this central database, all antidiabetic drugs prescribed with reimbursement and redeemed in pharmacies nationwide are regularly registered and, in addition, all hospital reports for inpatients and medical reports for out-patient visits are also recorded. This central database uses the patients’ social security numbers and collects data about patients’ diseases using codes of International Classification of Diseases (ICD), 10th version (ICD-10). The database covers close to 100% of the Hungarian population (9,957,731 subjects in 2012), therefore our analysis should be considered nationwide. The database has no information about private care visits which were marginal in Hungary in our investigation period.

We investigated retrospectively an 8-year long period from 2013 to 2020, inclusive. The period of 2013–2014 was used as reference in order to detect only the real starting therapy in 2015. Each person was followed until 31 December 2020 or until death (period of analysis: 2015–2020). 

Subjects with at least 2 ICD-10 codes of E10–E14 (beyond 30 days but within 365 days) were enrolled in our study. The date of the first appearance of the code was considered as the date of the diagnosis. Patients with only 1 ICD-10 code of E10–E14 were also included if they died within 60 days. Patients without these criteria but with at least 2 prescription redemptions of antihyperglycaemic agents (ATC A10 class) in two different days were also enrolled. Women with gestational diabetes (ICD-10: O2440) and polycystic ovary syndrome (ICD-10: E2820) were excluded from the analysis. 

For evaluating patterns of different therapeutic regimens, we evaluated treatment with novel and traditional antihyperglycaemic agents. As for novel antidiabetic drugs, 5 DPP-4 inhibitors (sitagliptin, vildagliptin, alogliptin, saxagliptin, and linagliptin) and their fix combinations with MET became available in 2008–2010, while GLP-1-RAs (exenatide, lixisenatide, liraglutide, semaglutide, and dulaglutide) and GLP-1-RAs + basal insulin analogue combinations (IDegLira, IGlarLixi) reached the market after 2010. Finally, SGLT-2 inhibitors (dapagliflozin, empagliflozin, ertugliflozin) and their fix combinations with MET became available from 2015 onwards. All novel antihyperglycaemic agents have the same (70%) reimbursement in Hungary. As for traditional antihyperglycaemic agents, five SUs (glibenclamide, gliclazide, gliquidone, glimepiride, glipizide) and MET (original and generic derivatives) are available in Hungary, all of them are reimbursed by 55%. As for treatment with INS, different preparations of both human insulin and insulin-analogues are on the market and reimbursed by 50–100%, depending on local rules for prescriptions. Thiazolidinediones (rosiglitazone, pioglitazone) were withdrawn from the market and, therefore, we did not evaluate this class of drugs. In Hungary, only MET and SU could be prescribed by family practitioners in primary care while patients should be referred to specialists (endocrinologists, diabetologists) for initiating INS or novel drugs (DPP-4 inhibitors, SGLT-2 inhibitors, GLP-1-RAs). 

Importantly, the reimbursement policy in our country did not change in the period of 2015–2020. Nevertheless, a small change was introduced in October 2014 when the reimbursement of a single generic metformin was discontinued due to financial reason. Consequently, new patients treated only with this generic metformin in monotherapy were not registered. 

For detecting the type of diabetes, we used a hierarchical classification algorithm for identifying subjects with type 1 diabetes mellitus (T1DM). In the database, patients were accounted for having T2DM if they did not meet criteria for T1DM (Table S1). 

Incidence cases are given as annual numbers of newly registered patients with T2DM (crude numbers). We calculated prevalence using annual number of patients with T2DM who were alive on the 1st of January in the particular year with previous registration with T2DM in the database. Newly detected (incident) cases of patients with T2DM in the particular year were also accounted for in the respective annual prevalence rate. In this way, we used period-prevalence, i.e., the sum of the incident and prevalent cases registered in the respective year (crude numbers). Prevalent T2DM cases based on prescription redemption of antidiabetic drugs were considered if at least one prescription redemption was registered in the respective year. 

We used the term of monotherapy if at least one prescription redemption of a single drug from the same antihyperglycaemic class was registered at least in one month per year. We used the term of combination therapy if at least two prescription redemptions of at least two different classes were registered at least in one month per year. 

In our analysis we focused on the treatment patterns with

- antihyperglycaemic agents (in monotherapy or in combination) in 2015–2020 (assessing prevalence of patients with different treatment regimes)
- antihyperglycaemic agents as initial therapy in 2015–2020 (assessing incidence of patients with initial therapy)
- novel drugs with cardiovascular/renal benefit (either in monotherapy or in combination) after initial therapy with MET or SU (assessing incidence of patients with such treatment after initial MET or SU therapy; for comparison, DPP-4-inhibitors and INS were also analysed).

Although we have detailed results of different treatment regimes, we only provide incidence/prevalence data of the top 5–10 treatment patterns (having the highest proportions) in this paper. 

In the tables, total number of patients means the sum of patients with social security numbers (each patient counts only once). We provide annual numbers of patients with the redeemed prescriptions of monotherapy or combination treatment between 2015 and 2020. In the figures, we illustrate their annual percentual occurrence among the total number of patients. Importantly, if a patient switched the therapy within a calendar year, he/she was attributed to both original and new treatment regimes, therefore the sum of annual numbers and that of percentual appearance may exceed 100% of total numbers of patients. In the text, prevalence/incidence rates of a given treatment means prevalence/incidence rates of patients with the respective treatment. 

We used linear regression analysis for evaluating trends in annual changes of treatment patterns. In supplementary tables, mean values and 95% confidence intervals are given. The level of significance was set at *p* < 0.05. 

As for the use of novel drugs with cardiovascular/renal benefit, we evaluated patients from the period of 2015–2019 and followed to 31 December 2020. In this analysis, Kaplan–Meier survival curves were generated to assess the proportion of patients who remained on initial therapy with MET or SU. 

Finally, a special condition should be mentioned. In March 2020, the Hungarian government ordered a lockdown due to coronavirus disease 2019 (COVID-19). This caused difficulties for patients in access to health care providers. 

Data protection met all requirements prescribed by General Data Protection Regulation (GDPR) as all data were anonymised at data extraction and we used non-identifiable aggregate data for comparing groups of patients. A study license number was needed and provided (IO43/35-3/2021) by the National Institute of Health Insurance Fund Management, Hungary. The study design was ethically approved by the Medical Research Council, Scientific and Research Committee, Budapest, Hungary (code number: BMEÜ/325-1/2022/EKU, date of approval: 4 July 2022). 

## 3. Results

**Figure 1:**
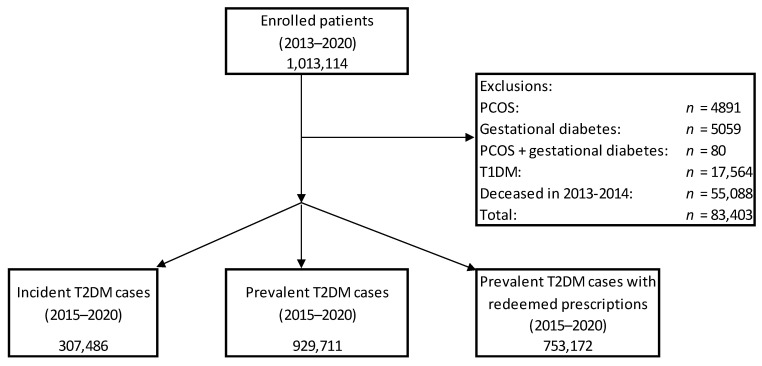
Flow-chart of the analysis (T1DM: type 1 diabetes mellitus, T2DM: type 2 diabetes mellitus, PCOS: polycystic ovary syndrome).

The total number of patients in the investigation period (2013–2020) was 1,013,114. After excluding patients with gestational diabetes, PCOS, T1DM, and death cases until 31 December 2014 (total number of excluded patients: *n* = 83,403), the number of patients in the period of 2015–2020 were as follows: incident cases: *n* = 307,486, prevalent cases: *n* = 929,711. We also determined the number of prevalent cases with prescription redemptions of antihyperglycaemic agents (*n* = 753,172) (Figure 1).

The incidence cases of T2DM continuously decreased from 2015 to 2019 but a greater decrease was observed thereafter, in 2020. On the other hand, prevalent cases steadily increased from 2015 to 2019 while there was a small decrease in 2020 (crude numbers in 2015: *n* = 682,274; in 2019: 760,032; in 2020: *n* = 752,367). The numbers of prevalent T2DM patients with redeemed prescriptions were about 20% smaller each year, compared to the number of total prevalent cases (Table 1). 

### 3.1. Prevalence of Patients with Different Treatment Patterns—Use of Antihyperglycaemic Agents in Monotherapy or in Combination between 2015 and 2020

The patterns of treatment with antihyperglycaemic agents changed over time. 

**Figure 2:**
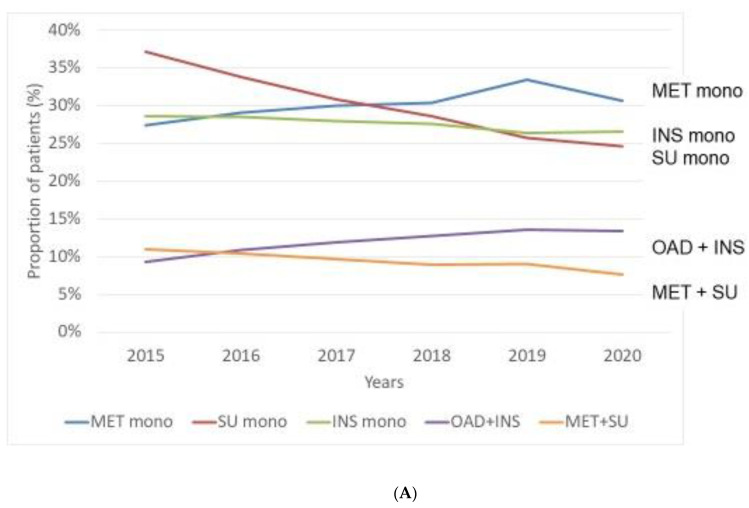

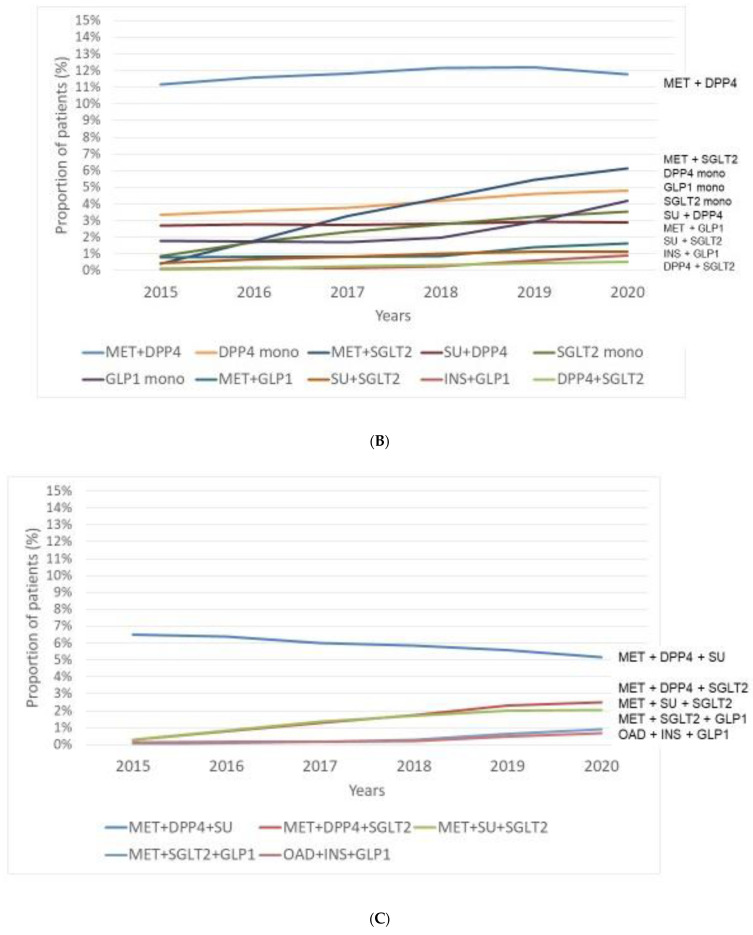
Treatment patterns of patients with type 2 diabetes mellitus (T2DM) in the period of 2015–2020. Prevalence of T2DM-patients treated with (**A**) traditional antihyperglycaemic agents in monotherapy or in combinations; (**B**) novel drugs in monotherapy or in dual combinations; (**C**) novel drugs in triple combinations. MET: metformin, INS: insulin, SU: sulfonylurea, OAD: oral antidiabetic drug, DPP4: dipeptidyl peptidase-4 inhibitor, SGLT2: sodium-glucose co-transporter-2 inhibitor, GLP1: glucagon-like peptide-1 receptor agonist, mono: monotherapy.

Regarding traditional treatment options, patients with MET monotherapy had the highest prevalence in 2015–2020, the proportion of patients with MET monotherapy increased continuously from 27% to 33% but a small decrease (31%) was observed in 2020, resulting in an insignificant annual change over time (*p* = 0.0553). Prevalence of INS monotherapy continuously and slightly decreased from 29% to 27% while that of SU monotherapy markedly decreased from 37% to 20%. Prevalence of dual combination with oral antidiabetic drug (OAD) + INS (OAD + INS) increased from 8% to 13%, while that of MET + SU dual combination decreased from 11% to 8% in this period (Figure 2A, Table 2 and Table S2). 

Novel drugs such as SGLT-2 inhibitors and GPL-1-RAs were less frequently used but use of DPP-4 inhibitors remained popular. Dual combination of MET + DPP-4 inhibitor proved to be a frequently used treatment option (11% in 2015 and 12% in 2020; resulting in an insignificant annual change over time; *p* = 0.0967). On the other hand, dual combination of MET + SGLT-2 inhibitor markedly increased over time (from <1% to 6%). Monotherapeutic use of DPP-4 inhibitors, SGLT-2 inhibitors, and GLP-1-RAs slightly increased (from 3% to 5%, from 1% to 4%, and from 2% to 4%, respectively). Dual combination therapy with SU + DPP 4-inhibitor and that of MET + GLP-1-RA slightly increased (reaching 3% and 2%, respectively, in 2020). All other dual combination treatment (SU + SGLT-2 inhibitor, INS + GLP-1-RA, DPP-4 inhibitor + SGLT-2 inhibitor) had around 1% proportion with increasing trend among treated patients (Figure 2B, Table 2 and Table S2). 

As for novel drugs in triple combination therapy, use of MET + DPP-4 inhibitor + SU proved to be the most popular, but its use slightly but significantly decreased over time from 6% to 5%. On the other hand, a slight increase in the use of MET + DPP-4 inhibitor + SGLT-2 inhibitor and that of MET + SU + SGLT-2 inhibitor combination was observed (from <1% to 2%). Although the use of triple combination of MET + SGLT-2 inhibitor + GLP-1-RA and that of OAD + INS + GLP-1-RA slightly increased, their use did not exceed 1% proportion in the patient’s group (Figure 2C, Table 2). 

Some patients were treated with quadruple oral therapy (MET + SU + DPP-4 inhibitor + SGLT-2 inhibitor); the annual number of patients increased from 840 (<1%) to 5530 (1%), from 2015 to 2020. 

### 3.2. Incidence of Patients with Initial Therapy—Use of Antihyperglycaemic Agents as Initial Therapy in the Period of 2015–2020 

**Figure 3:**
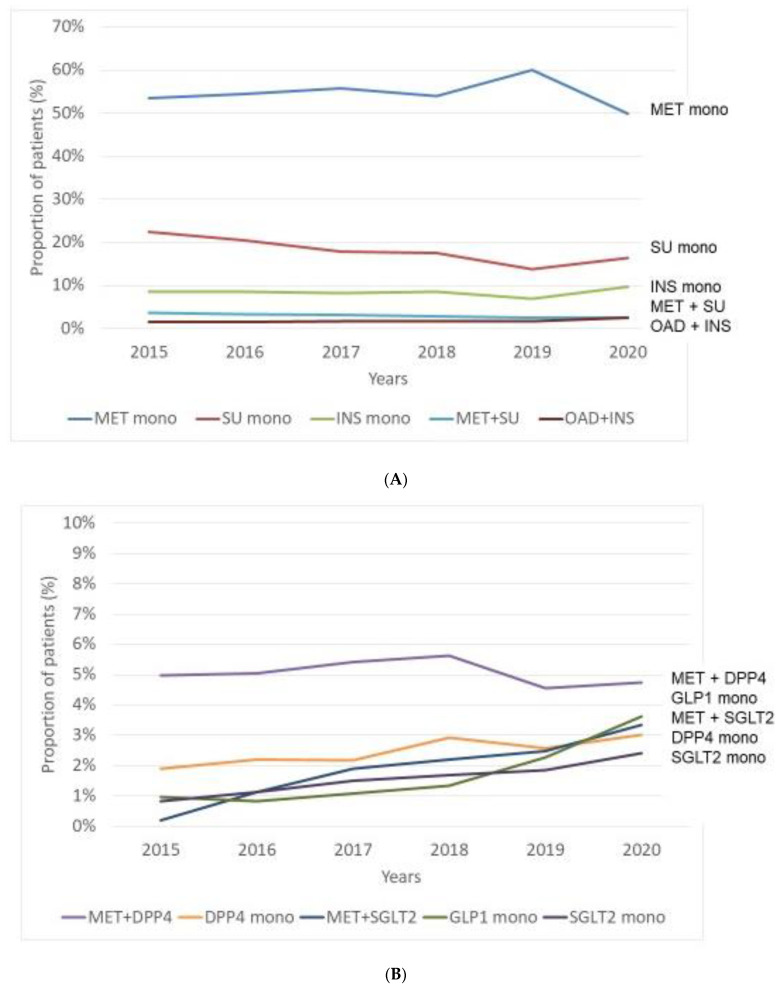
Treatment patterns of patients with type 2 diabetes mellitus (T2DM) in the period of 2015–2020, according to the initial pharmacological intervention. Top 10 incidence rates of T2DM-patients treated initially with traditional antihyperglycaemic agents (**A**) or with novel drugs (**B**). MET: metformin, INS: insulin, SU: sulfonylurea, OAD: oral antidiabetic drug, DPP4: dipeptidyl peptidase-4 inhibitor, SGLT2: sodium-glucose co-transporter-2 inhibitor, GLP1: glucagon-like peptide-1 receptor agonist, mono: monotherapy.

Regarding traditional therapies, MET remained the leading initial antihyperglycaemic agent; we observed a continuous increase in incidence of patients with MET monotherapy from 53% to 60% between 2015 and 2019, however, a slight decrease (50%) was documented thereafter in 2020, resulting in an insignificant annual change over the entire time. Although incidence of patients with SU treatment decreased significantly between 2015 and 2020 (from 22% to 16%), it remained the second most frequently used initial monotherapy. Incidence of patients with INS monotherapy did not change significantly over the entire period (9–10%). Interestingly, dual combinations as initial treatment (MET + SU, OAD + INS) were also observed in some patients, with a decreasing trend in the former and with an increasing trend in the latter treatment pattern (Figure 3A, Table 3 and Table S3). 

Novel drugs were less frequently used as initial therapy. Incidence of patients with dual combination of MET + DPP-4 inhibitors was the highest but remained unchanged (5%). Incidence of patients with monotherapy with GLP-1-RA increased from 1% to 4%, that of DPP-4 inhibitors from 2% to 3% and that of SLGT-2 inhibitors from 1% to 2%. Incidence of patients with dual combination of MET + SGLT-2 inhibitors exhibited a continuous increase over time from <1% to 3% (Figure 3B, Table 3 and Table S3).

### 3.3. Incidence (Appearance) of Novel Drugs with Cardiovascular/Renal Benefit (Either in Monotherapy or in Combination) after Initial Therapy with MET or SU 

**Figure 4:**
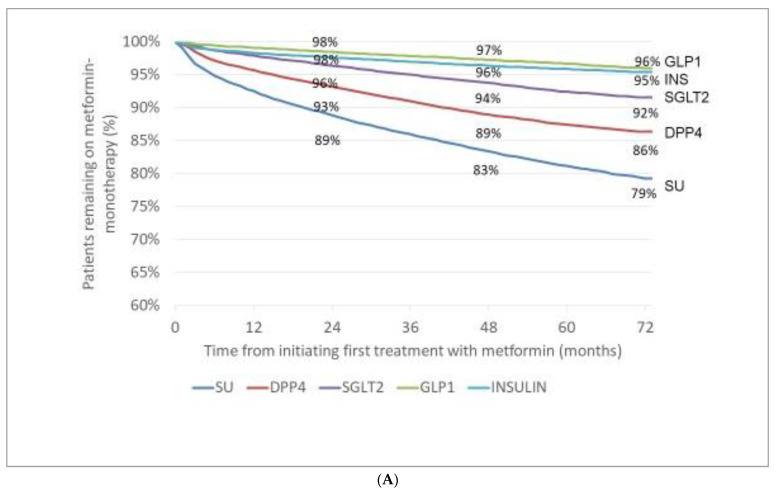

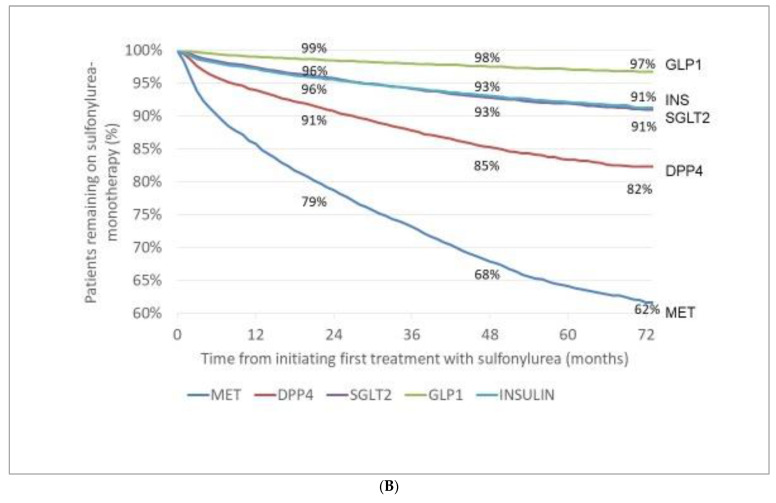
Incidence (appearance) of add-on treatment (either in monotherapy or in combination) after initiating first treatment with metformin (**A**) or sulfonylurea (**B**). MET: metformin, SU: sulfonylurea, DPP4: dipeptidyl peptidase-4 inhibitor, SGLT2: sodium-glucose co-transporter-2 inhibitor, GLP1: glucagon-like peptide-1 receptor agonist.

The number of patients with initial MET monotherapy was 113,256. Novel drugs with cardiovascular/renal benefit were less frequently added than other drugs in the subsequent six year after MET initial monotherapy (incidence rates of patients with GLP-1-RAs: 2%, 3%, and 4%; with SGLT-2 inhibitors: 4%, 6%, and 8%; for comparison incidence rates with DPP-4 inhibitors: 7%, 11%, and 14%; with SU: 11%, 17%, and 21%; with INS: 2%, 4%, and 5%, at months 24, 48, and 72, respectively) (Figure 4A).

The number of patients with initial SU monotherapy was 37,596. Novel drugs with cardiovascular/renal benefit were less frequently added than other drugs in the subsequent six year after SU initial monotherapy (incidence of patients with GLP-1-RAs: 1%, 2%, and 3%; with SGLT-2-inhibitors: 4%, 7%, and 9%; for comparison incidence rates with DPP-4 inhibitors: 9%, 15%, and 18%; with MET: in 11%, 32%, and 38%; with INS: 4%, 7%, and 9%, at months 24, 48, and 72, respectively) (Figure 4B).

## 4. Discussion

In our nationwide analysis, we documented important changes in treatment patterns with antihyperglycaemic agents in patients with T2DM from 2015 to 2020.

- Regarding prevalence of patients with traditional antidiabetic drugs, we observed a relatively stable prevalence of patients with MET reaching the highest proportion among monotherapy in 2020. On the other hand, prevalent cases with SUs markedly and those of INS slightly decreased over time. Among novel drugs, DPP-4 inhibitors were the most frequently administered drugs. Although we found a clearly increasing rate of patients with SGLT-2 inhibitors and GLP-1-RAs, these drugs were used only in a small proportion of patients.
- As for initial therapy, patients with MET monotherapy had the highest incidence rate, while SUs—despite their steadily diminishing use—remained the second most frequently chosen initial monotherapy; incidence of patients with INS monotherapy did not change significantly over the entire period.
- After initial therapy with MET or SU, the incidence rates of patients with add-on GLP-1-RAs and those of add-on SGLT-2 inhibitors slowly increased in a relatively narrow range in the subsequent six years.
- Some abrupt alterations in the 6-year-long trends were observed in 2020, probably due to increasing rate of COVID-19 and the closedown in Hungary (from March 2020 onwards).

In our study we used a nationwide database to investigate changes in treatment patterns with different antihyperglycaemic drugs in patients with T2DM. It is accepted that pharmacy claims registries are reliable sources of data based on prescription refills. Using such database, meaningful results can be obtained which are of importance for both health care providers and health authorities [15]. 

Investigating prevalence and incidence rates of patients with different treatment patterns, we provided data on the use of antihyperglycaemic agents in patients with T2DM. This method should be considered more clinically oriented than other analyses based on utilization and expenditure data of antihyperglycaemic agents [16]. 

The incidence rate of total number of patients with T2DM decreased over time, however, a strong decline was observed in 2020. In addition, the steady increase in prevalence rate of total number of patients with T2DM was halted in 2020 (Table 1). While the trends in 2015–2019 are consistent with our former and other observations [6,7], it is very likely that the steep decline in incidence rate and the halted increase in prevalence rate could be attributed to COVID-19 pandemic [17]. The COVID-19 lockdown in Hungary was ordered in March 2020 onwards. As a consequence, the access to health care providers became difficult and limited. Although the widespread increase of telemedicine slowly counterbalanced this drawback in diabetes management [18], the impact of COVID-19 pandemic on treatment patterns should be considered as an important factor affecting therapeutic practice in patients withT2DM. 

In our analysis we assessed a 6-year-long period from 2015 to 2020. Beside traditional drugs (MET, SU, INS) for treating patients with T2DM, novel, innovative drugs became available 15 years ago. DPP-4 inhibitors reached the market first, GLP-1-RAs and SGLT-2 inhibitors arrived later. Importantly, all novel antihyperglycaemic drugs were available in Hungary during the entire observational period. Nevertheless, the timeframe of availability of novel antidiabetic drugs differed and might affect their use in practice. 

Among traditional antihyperglycaemic agents, we analysed the changes in trends of treatment patterns with MET, SU, and INS.

MET should be considered as first line pharmacological treatment when glycaemic target cannot be achieved by life-style modification only [13,19]. Although this widely accepted recommendation has been recently challenged by some authors and cardiological guidelines [20], our results confirmed the high acceptance of MET both in monotherapy and in combinations (Figure 2A) and also in initial treatment (Figure 3A). This is partly due to the fact that MET has a long history and diabetologists could gain huge experiences with its use [21]. In addition, its generic forms became available and widely affordable. It is noteworthy that some new data regarding the role of MET in patients with T2DM and beyond (cardiovascular benefit, cancer prevention, treating interstitial pulmonary fibrosis, affecting microbiome, benefit in viral infections including COVID-19, influencing ageing) hold the promise for its further, undiminished use [22,23,24,25,26,27,28].

SUs belong to another traditional antidiabetic class of treatment [29]. Although derivatives of this class have special characteristics with clinically relevant differences, they have unfavourable side-effects (increasing risk of hypoglycaemia, weight gain) in general. In addition, their cardiovascular safety has been challenged, but clearly, they have no cardiovascular or renal benefit. They are easily affordable in Hungary. In our study, we observed a steadily diminishing use of SUs in clinical practice (Figure 2A) and also in initial treatment (Figure 3A); the decreasing trend is in line with its recent positioning in a modern treatment algorithm for patients with T2DM [30].

INS has been a well-established treatment option even for patient with T2DM. Although treatment with INS has a 100-year-long history [31], the armamentarium of treatment with INS entirely renewed by the availability of insulin analogues some decades ago [32]. T2DM with acute metabolic decompensation is an absolute indication for initiating INS treatment. In our study, prevalence of treatment pattern with INS proved to be slightly decreased (Figure 2A) while incidence as initial treatment was relatively stable over time (Figure 3A). In general, we can expect that we will not use INS in the future as frequently as some years ago in T2DM patients. It is simply due to its characteristics (subcutaneous administration, producing weight gain, and increasing risk of hypoglycaemia). Importantly, GLP-1-RAs and SGLT-2 inhibitors recently became preferable drugs due to their disease modifying nature in most patients with T2DM [33]. In addition, de-escalation of treatment with INS became especially important among elderly patients with T2DM [34,35]. 

DPP-4 inhibitors have the oldest availability among novel, innovative drugs in Hungary for treating patients with T2DM. According to our data, these drugs are still popular among physicians (Figure 2B,C). Although they proved to be safe, cardiovascular or renal benefits were not documented in large, randomised, controlled trials [36]. As a result, recommendations for using DPP-4-inhibitors substantially changed very recently [37,38] which was not really mirrored in numbers between 2015 and 2020 in our study. 

In our analysis, we had a special interest in investigating changes of treatment patterns with antihyperglycaemic agents having cardiovascular/renal benefits. According to recent guidelines, both GLP-1-RAs and SGLT-2 inhibitors should be preferred for patients with T2DM and high cardiovascular/renal risk [13,19,39,40,41]. In line with other observations [42], we observed a clear and steady increase in the use of these drugs in our entire cohort, however, these two classes of drugs were chosen with a delay and only in a small proportion of patients as add-on treatment to MET or SU initial monotherapy (Figure 4). The results of the international CAPTURE study with adult T2DM patients (*n* = 9823) clearly indicated a gap between clinical benefit evidence and real-world practice; GLP-1-RAs and/or SGLT-2 inhibitors were used only a small portion (21.9%) of patients, which was similar in participants with and without cardiovascular diseases (21.5% and 22.2%, respectively) [42]. Unfortunately, explanation for this phenomenon could not be retrieved from our database; however, the clinical inertia and its disadvantageous consequences are well documented in T2DM patients after initial MET monotherapy [43]. 

T2DM has multifactorial origin with different pathways to reach clinical manifestation; therefore, the use of antihyperglycaemic drugs in combinations has strong theoretical background [44]. Most of the clinical guidelines recommend stepwise combination strategy after initial treatment with MET [13]. However, timing is essential. In the VERIFY clinical trial, early intervention with a double combination therapy (MET + DPP-4 inhibitor vildagliptin) provided greater and more durable long-term benefits compared with initial MET monotherapy in traditional stepwise approach [45]. In our study, MET in double combination with SU, INS, DPP-4 inhibitors, SGLT-2 inhibitors, or GLP-1-RAs was relatively often used while we found triple combination regimes less frequently (Figure 2 and Figure 3). Interestingly, we picked up prevalent cases with quadruple oral treatment as well, that might occur in the real-world practice [46]. 

Taken together, our results are consistent with recent similar registry-based surveys from different countries in Europe (Sweden, Greece, Italy, Denmark, Romania) [47,48,49,50,51,52].

Our results have to be interpreted within the context of their limitations. In our study no specific data were available about important covariates such as severity of the disease, comorbidities, glycaemic control, socioeconomic status, or incidence of side-effects of ongoing antidiabetic treatment. While we acknowledge the importance of these cofounders in diabetes management, we feel that our results are meaningful for characterizing the main changes of antihyperglycaemic treatment in patients with T2DM. For classification of existing diabetes for T2DM, we used a hierarchical classification algorithm; although it was carefully designed, some non-differential misclassification could not be ruled out. This may be especially relevant regarding LADA (Latent Autoimmune Diabetes in Adults) and MODY (Maturity Onset Diabetes in the Young) cases. Nevertheless, having a high number of patients enrolled, the impact of this potential misclassification should be considered minimal. The generalizability of our results is limited because the healthcare system and access to different treatment options are country-specific. In our analysis, we could not investigate the prescribing patterns according to the qualifications of physicians. 

Regarding strengths of the study, it is noteworthy that our investigation was based on a nationwide database. Additionally, all traditional and novel antihyperglycaemic agents are reimbursed (one exception: some generic forms and extended-release MET) and the reimbursement policy proved to be stable over the last 15 years in Hungary.

## 5. Conclusions

In the period of 2015–2020, the documented changes in antihyperglycaemic treatment patterns in patients with T2DM followed the new scientific recommendations but remained below our expectations with respect to timing and magnitude. Therefore, we assess the observed changes as initial steps in improvement of pharmacological treatment approach of T2DM patients. To achieve further results, continuous efforts should be made to implement new agents with cardiovascular/renal benefits into therapeutic management in time, in a much larger proportion of adult T2DM population, and without delay.

## Figures and Tables

**Table 1:** Patients with type 2 diabetes mellitus (T2DM): incident and prevalent cases (crude numbers) in the central database between 2015 and 2020 (reference period: 2013–2014).

	Incident Cases (*n*)	Prevalent Cases (*n*)	Prevalent Cases with Redeemed Prescriptions of Antidiabetic Drugs (*n*)
2015	60,049	682,274	526,076
2016	57,726	706,617	535,220
2017	54,059	727,071	541,072
2018	52,506	743,585	543,494
2019	53,279	760,032	560,142
2020	29,865	752,367	540,607
2015–2020	307,486	929,711	753,172

**Table 2:** Number of prevalent T2DM patients (*n*) treated with traditional or novel antihyperglycaemic agents in monotherapy or in combinations between 2015 and 2020.

	2015	2016	2017	2018	2019	2020
Total number of patients with redeemed prescriptions	526,076	535,220	541,072	543,494	560,142	540,607
Number of patients with traditional agents (monotherapy or combination)						
MET monotherapy	144,044	155,563	162,311	165,088	187,110	165,779
INS monotherapy	150,659	152,503	151,282	149,983	147,666	143,500
SU monotherapy	195,355	181,033	166,961	155,466	144,375	133,283
OAD + INS combination	48,916	58,037	64,580	69,059	75,875	72,357
MET + SU	57,918	55,566	52,230	48,497	50,596	41,428
Number of patients with novel agents (monotherapy or dual combination)						
MET + DPP-4 inhibitor	58,700	62,108	63,934	66,083	68,331	63,734
MET + SGLT-2 inhibitor	2148	9578	17,770	23,640	30,544	33,144
DPP-4 inhibitor	17,696	19,170	20,476	22,840	25,907	25,940
GLP-1-RAs	9349	9305	9357	10,717	16,495	22,683
SGLT-2- inhibitor	4651	9121	12,579	15,087	18,150	19,163
SU + DPP-4 inhibitor	14,226	14,790	14,846	15,306	16,417	15,746
MET + GLP-1-RA	4136	4367	4427	4634	7395	8541
SU + SGLT-2 inhibitor	2286	3640	4489	5297	5968	6039
INS + GLP-1-RA	673	926	833	1374	3172	4745
DPP-4 inhibitor + SGLT-2 inhibitor	323	838	1381	1816	2434	2870
Number of patients with novel agents (triple combination)						
MET + DPP-4 inhibitor + SU	34,136	34,113	32,575	31,833	31,282	27,948
MET + DPP-4 inhibitor + SGLT-2 inhibitor	1499	4104	6705	9098	12,133	13,078
MET + SU + SGLT-2 inhibitor	1438	4237	7118	8974	10,555	10,751
MET + SGLT-2 inhibitor + GLP-1-RA	171	486	944	1588	3261	4673
OAD + INS + GLP-1-RA	652	900	850	1081	2539	3531

MET: metformin, INS: insulin, SU: sulfonylurea, OAD: oral antidiabetic drug, DPP-4: dipeptidyl peptidase-4. SGLT-2: sodium-glucose co-transporter-2, GLP-1-RA: glucagon-like peptide-1 receptor agonist.

**Table 3:** Incidence of T2DM patients (*n*) with initial antihyperglycaemic agents between 2015 and 2020. (Top 10 treatment patterns with the highest incidence).

	2015	2016	2017	2018	2019	2020
Total number of patients with initial redeemed prescriptions	43,115	42,023	39,236	37,250	42,151	25,496
Number of patients with initial traditional agents (monotherapy or combination)						
MET monotherapy	23,060	22,901	21,889	20,128	25,278	12,710
SU monotherapy	9686	8611	7006	6512	5781	4194
INS monotherapy	3712	3599	3197	3203	2916	2481
MET + SU	1587	1376	1209	1054	1065	653
OAD + insulin	622	655	653	650	728	631
Number of patients with initial novel agents (monotherapy or combination)						
MET + DPP-4 inhibitor	2149	2119	2125	2095	1924	1208
GLP-1-RA	412	346	427	502	953	921
MET + SGLT-2 inhibitor	85	470	745	822	1045	851
DPP-4 inhibitor	814	926	857	1086	1089	769
SGLT-2- inhibitor	358	470	590	631	781	616

MET: metformin, INS: insulin, SU: sulfonylurea, OAD: oral antidiabetic drug, DPP-4: dipeptidyl peptidase-4. SGLT-2: sodium-glucose co-transporter-2, GLP-1-RA: glucagon-like peptide-1 receptor agonist.

## Data Availability

The database of the National Institute of Health Insurance Fund Management (Hungary) can be accessed for research on reasonable request. The datasets generated and/or analysed during the current study are available from G.R. and Z.A.-T. on reasonable request.

## Supplementary Materials

The following supporting information can be downloaded at: https://www.mdpi.com/article/10.3390/medicina58101382/s1, Table S1: Method for identification of subjects with Type 2 diabetes in the central database; Table S2: Annual changes in proportion (%) of prevalent T2DM patients treated with traditional or novel antihyperglycaemic agents in monotherapy or in combinations between 2015 and 2020; Table S3. Annual changes in proportion (%) of incident T2DM patients with initial antihyperglycaemic agents between 2015 and 2020.
