# Supplementary material for: Changing Patterns of Antihyperglycaemic Treatment among Patients with Type 2 Diabetes in Hungary between 2015 and 2020—Nationwide Data from a Register-Based Analysis
Source: Medicina (Kaunas). 2022 Oct 1;58(10):1382. doi: 10.3390/medicina58101382 (PMC9612371; doi:10.3390/medicina58101382)
Supplement: Supplementary file 1 [file medicina-58-01382-s001.zip › medicina-1884055-supplementary.pdf]

Supplementary material

Table S1

Method for identification of subjects with Type 2 diabetes in the central database

Subjects

All patients had a special social security number. Diseases were qualified by using ICD codes (International Classification of Diseases, 10th version). Women with gestational diabetes (ICD-10: O2440) and those with polycystic ovary syndrome (ICD-10: E2820) were excluded from the analysis.

Patients with type 1 or type 2 diabetes were investigated (type 1 ICD-10: E10, type 2 ICD-10: E11). First, we identified patients with type 1 diabetes, and then, patients not having type 1 diabetes were considered as having type 2 diabetes.

For identification of subjects with type 1 diabetes a hierarchical algorithm with 8 steps were used. There was a basic definition and 7 further, hierarchical, stepwise definitions. Identification process was stopped, and type 1 diabetes was determined if the basic definition or the first hierarchical definition and criterion at step 6 fulfilled.

Basic definition:

Patients had type 1 diabetes, if he/she had in the registry

- ICD code E10, and
- the ratio of E10/(E10+E11) code was ≥50%, and
- insulin was prescribed for the patient, and
- no oral antidiabetic drug was prescribed 180 days after the first prescription of insulin

Hierarchical, stepwise definitions:

Patients also had type 1 diabetes, if the following criteria fulfilled

Step 1:

- ICD code E10, and
- the ratio of E10/(E10+E11) code was ≥50%, and
- insulin was prescribed for the patient, and
- 180 days after the first prescription of insulin, minimum 6 prescription redemptions of insulin and maximum 3 prescription redemptions of oral antidiabetic drugs occurred

Step 2:

- no ICD E10 or E11 codes, and
- insulin was prescribed for the patient, and
- 180 days after the first prescription of insulin, minimum 6 prescription redemptions of insulin and maximum 3 prescription redemptions of oral antidiabetic drugs occurred, and
- no antihyperglycaemic agent was used in 2013 and the patient age was <35 years at the first prescription

Step 3:

- ICD E10 code, and

- the ratio of E10/(E10+E11) code was ≥50%, and
- the first prescription was insulin followed by insulin prescriptions exclusively, within 180 days, and
- prescriptions of oral antidiabetic drugs might occur even beyond 180 days but should be accompanied by insulin prescriptions within ±7 days

Step 4:

- ICD E10 code, and
- the ratio of E10/(E10+E11) code was ≥50%, and
- the first prescription was insulin followed by insulin prescriptions exclusively, within 180 days, and
- prescriptions of oral antidiabetic drugs might occur even beyond 180 days not accompanied by insulin prescriptions, but the last prescription of oral drug was followed at least one insulin prescription

Step 5:

- ICD E10 code, and
- the ratio of E10/(E10+E11) code was ≥50%, and
- the first prescription was insulin followed by insulin prescriptions exclusively, within 180 days, and
- prescriptions of oral antidiabetic drugs might occur even beyond 180 days with or without insulin prescription within ±7 days but the last prescription should be followed by at least one insulin prescription within ±6 days

Patients who did not meet the basic or the hierarchical, stepwise criteria for type 1 diabetes were considered as having type 2 diabetes.

Step 6:

Patients with age of >40 years at the diagnosis and having type 1 diabetes according to the above-mentioned criteria were re-classified as having type 2 diabetes.

Step 7:

Patients without any of the above-mentioned criteria were classified as having type 2 diabetes.

Table S2. Annual changes in proportion (%) of prevalent T2DM patients treated with traditional or novel antihyperglycaemic agents in monotherapy or in combinations between 2015 and 2020

	Mean annual change (%)	95% CI	p value
**Patients with traditional agents (monotherapy or combination)**			
**MET monotherapy**	0.8518	-0.0309 1.7345	0.0553
**INS monotherapy**	-0.4923	-0.7054 -0.2792	**0.0030**
**SU monotherapy**	-2.5372	-2.9899 -2.0845	**0.0001**
**OAD + INS combination**	0.8373	0.4797 1.1950	**0.0029**
**MET + SU**	-0.6145	-0.8182 -0.4108	**0.0011**
**Patients with novel agents (monotherapy or dual combination)**			
**MET + DPP-4 inhibitor**	0.1490	-0.0424 0.3404	0.0967
**MET + SGLT-2 inhibitor**	1.1623	0.9730 1.3515	**0.0001**
**DPP-4 inhibitor**	0.3063	0.2468 0.3659	**0.0001**
**GLP-1-RAs**	0.4558	0.0712 0.8405	**0.0302**
**SGLT-2 inhibitor**	0.5246	0.4060 0.6433	**0.0003**
**SU + DPP-4 inhibitor**	0.0462	0.0215 0.0709	**0.0066**
**MET + GLP-1-RA**	0.1701	0.0466 0.2936	**0.0187**
**SU + SGLT-2 inhibitor**	0.1442	0.0938 0.1946	**0.0014**
**INS + GLP-1-RA**	0.1501	0.0474 0.2528	**0.0154**
**DPP-4 inhibitor + SGLT-2 inhibitor**	0.0975	0.0923 0.1027	**<0.0001**
**Patients with novel agents (triple combination)**			
**MET + DPP-4 inhibitor + SU**	-0.2607	-0.3172 -0.2043	**0.0002**
**MET + DPP-4 inhibitor + SGLT-2 inhibitor**	0.4582	0.3900 0.5265	**<0.0001**
**MET + SU + SGLT-2 inhibitor**	0.3659	0.2374 0.4944	**0.0014**
**MET + SGLT-2 inhibitor + GLP-1-RA**	0.1710	0.0970 0.2449	**0.0030**
**OAD + INS + GLP-1-RA**	0.1061	0.0322 0.1801	**0.0163**

MET: metformin, INS: insulin, SU: sulfonylurea, OAD: oral antidiabetic drug, DPP-4: dipeptidyl peptidase-4

SGLT-2: sodium-glucose co-transporter-2, GLP-1-RA: glucagon-like peptide-1 receptor agonist, CI: confidence interval

Table S3. Annual changes in proportion (%) of incident T2DM patients with initial antihyperglycaemic agents between 2015 and 2020

	Mean annual change (%)	95% CI		p value
**Patients with initial traditional agents (monotherapy or combination)**				
**MET monotherapy**	-0.1000	-2.5432	2.3431	0.9150
**SU monotherapy**	-1.4509	-2.5173	-0.3846	**0.0195**
**INS monotherapy**	0.0320	-0.6415	0.7054	0.9016
**MET + SU**	-0.2312	-0.3091	-0.1534	**0.0012**
**OAD + INS combination**	0.1642	0.0194	0.3090	**0.0346**
**Number of patients with novel agents (monotherapy or dual combination)**				
**MET + DPP-4 inhibitor**	-0.0702	-0.3510	0.2102	0.5252
**GLP-1-RA**	0.5101	0.1413	0.8791	**0.0185**
**MET + SGLT-2 inhibitor**	0.5740	0.4132	0.7350	**0.0185**
**DPP-4 inhibitor**	0.2146	0.0682	0.3611	**0.0152**
**SGLT-2 inhibitor**	0.2949	0.2249	0.3649	**0.0003**

MET: metformin, INS: insulin, SU: sulfonylurea, OAD: oral antidiabetic drug, DPP-4: dipeptidyl peptidase-4

SGLT-2: sodium-glucose co-transporter-2, GLP-1-RA: glucagon-like peptide-1 receptor agonist, CI: confidence interval
